# A Method for Genetically Installing Site-Specific Acetylation in Recombinant Histones Defines the Effects of H3 K56 Acetylation

**DOI:** 10.1016/j.molcel.2009.07.027

**Published:** 2009-10-09

**Authors:** Heinz Neumann, Susan M. Hancock, Ruth Buning, Andrew Routh, Lynda Chapman, Joanna Somers, Tom Owen-Hughes, John van Noort, Daniela Rhodes, Jason W. Chin

**Affiliations:** 1 Medical Research Council Laboratory of Molecular Biology, Hills Road, Cambridge CB2 0QH, England, UK; 2 Physics of Life Processes, Leiden Institute of Physics, Leiden University, The Netherlands; 3 Wellcome Trust Centre for Gene Regulation and Expression, School of Life Sciences, University of Dundee, Dundee DD1 5EH, Scotland, UK

**Keywords:** DNA, PROTEINS

## Abstract

Lysine acetylation of histones defines the epigenetic status of human embryonic stem cells and orchestrates DNA replication, chromosome condensation, transcription, telomeric silencing, and DNA repair. A detailed mechanistic explanation of these phenomena is impeded by the limited availability of homogeneously acetylated histones. We report a general method for the production of homogeneously and site-specifically acetylated recombinant histones by genetically encoding acetyl-lysine. We reconstitute histone octamers, nucleosomes, and nucleosomal arrays bearing defined acetylated lysine residues. With these designer nucleosomes, we demonstrate that, in contrast to the prevailing dogma, acetylation of H3 K56 does not directly affect the compaction of chromatin and has modest effects on remodeling by SWI/SNF and RSC. Single-molecule FRET experiments reveal that H3 K56 acetylation increases DNA breathing 7-fold. Our results provide a molecular and mechanistic underpinning for cellular phenomena that have been linked with K56 acetylation.

## Introduction

The posttranslational acetylation of chromatin on the ɛ-amine of lysine residues in histone proteins defines the epigenetic status of human embryonic stem cells and is a crucial regulator of DNA replication, chromosome condensation, transcription, and DNA repair in model organisms (Grunstein, 1997; Jenuwein and Allis, 2001; Kouzarides, 2007; Peterson and Laniel, 2004; Shahbazian and Grunstein, 2007; Sterner and Berger, 2000; Xie et al., 2009). Acetylation may alter nucleosome or chromatin structure and function directly or may act to recruit other factors to the genome (Jenuwein and Allis, 2001; Kouzarides, 2007) via interaction with bromodomain-containing proteins (Yang, 2004) and other potential acetyl-lysine binding modules (Li et al., 2008).

It is emerging that modifications in the globular core of histones play crucial roles in regulating the structure and function of chromatin and controlling biological function (Cosgrove et al., 2004). H3 K56 acetylation is a particularly important modification in the globular core of H3 (Masumoto et al., 2005; Ozdemir et al., 2005; Xu et al., 2005) that is conserved from yeast to humans (Garcia et al., 2007). Numerous reports have demonstrated its role in DNA repair and replication, regulation of transcription, and chromatin assembly (Celic et al., 2006, 2008; Chen et al., 2008; Das et al., 2009; Driscoll et al., 2007; Han et al., 2007; Hyland et al., 2005; Li et al., 2008; Ozdemir et al., 2005; Rufiange et al., 2007; Williams et al., 2008; Xie et al., 2009; Xu et al., 2005, 2007; Yang et al., 2008). K56 is located on the first α helix of H3 and makes a water-mediated contact close to the DNA at the entry-exit point on the nucleosome (Davey et al., 2002). This has led to the proposal that K56 acetylation modulates the binding of the DNA in the nucleosome. Though H3 K56 acetylation is clearly important in defining epigenetic status and regulating transcription, replication, and repair, it has not been possible to experimentally and quantitatively test the mechanistic proposals for how K56 acetylation might affect these complex cellular phenomena (Celic et al., 2006; Chen et al., 2008; Cosgrove et al., 2004; Driscoll et al., 2007; Han et al., 2007; Hyland et al., 2005; Li et al., 2008; Masumoto et al., 2005; Rufiange et al., 2007; Xu et al., 2005; Xu et al., 2007) because it has not been possible to synthesize homogeneously acetylated H3 K56.

Current methods to introduce site-specific acetylation into recombinant histones include enzymatic posttranslational modification and native chemical ligation (McGinty et al., 2008; Shogren-Knaak et al., 2006). Core histones consist of a structural domain and a long N-terminal tail, and chemical ligation provides an approach suitable for introducing modifications into histone tails. For example, the role of H4 K16 acetylation in antagonizing chromatin compaction was demonstrated with histones modified at the N-terminal tail produced by native chemical ligation (Shogren-Knaak et al., 2006). This method remains challenging, as it requires the synthesis of large quantities of peptide thioester, has not been demonstrated for acetylation in the structured core of histones, and yields small quantities of acetylated protein. Enzymatic posttranslational acetylation of histones has also proved challenging (Robinson et al., 2008). The acetyl-transferase enzymes are large complexes that are difficult to purify. Moreover, enzymatic acetylation does not yield homogeneous samples, as acetylation at the desired site is not quantitative and rarely exceeds 30%, and in vitro acetylation at other sites leads to heterogeneous samples (Robinson et al., 2008). We recently reported proof-of-principle experiments in which we demonstrated the production of a homogeneously acetylated protein using an aminoacyl-tRNA synthetase and tRNA_CUA_ pair that we created by directed evolution. This pair directs the incorporation of acetyl-lysine in response to an amber codon in the gene of interest encoded on a plasmid in *E*. *coli* (Neumann et al., 2008). While this system is, in principle, applicable to the production of homogeneously and site-specifically acetylated histones, its utility has previously only been demonstrated in a single case with MnSOD, a small nonhistone enzyme.

We report an improved method for genetically encoding site-specific and homogeneous acetylation of histones in *E. coli*. This method has permitted the production of histones modified in the core of the protein, enabling us to produce recombinant histone H3 that is acetylated on K56 in milligram quantities. We have assembled histone octamers, nucleosomes, and chromatin arrays that bear this modification, and we have investigated the effect of H3 K56 acetylation on nucleosome and chromatin structure and function. We have examined the effect of H3 K56 acetylation on nucleosome stability, transient unwrapping of DNA from single nucleosomes, chromatin compaction, and nucleosome remodeling by SWI/SNF and RSC to test the mechanistic hypotheses on the role of this modification that have been proposed on the basis of cellular experiments.

## Results

### Production of Site-Specifically Acetylated Histones

**Figure 1:**
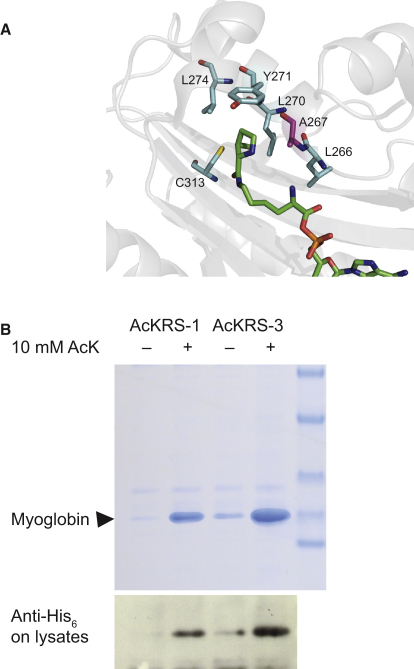
Selection of an Improved Acetyl-Lysyl-tRNA Synthetase/tRNA_CUA_ Pair for the Incorporation of Acetyl-Lysine in Recombinant Proteins (A) The active site of *M. mazei* PylRS bound to pyrrolysine (figure created using PyMOL [http://www.pymol.org] and PDB file 2Q7H). The residues mutated relative to the wild-type sequence are shown as sticks. Residues in cyan are mutated in the progenitor AcKRS-1 and were randomized again in the new library; A267 (magenta) was only included in the new library. (B) Characterization of a more efficient acetyl-lysyl-tRNA synthetase/tRNA_CUA_ pair. Myoglobin-His_6_ was expressed in *E. coli* DH10B from pMyo4TAG PylT (Neumann et al., 2008) (containing a hexa-histidine-tagged myoglobin gene with an amber codon at position 4 and the gene encoding *Mb*tRNA_CUA_) in the presence or absence of 10 mM acetyl-lysine using either pBK AcKRS-1 or pBK AcKRS-3. The proteins were purified by Ni^2+^ chromatography and analyzed by 4%–12% SDS-PAGE or detected in total lysates by western blot with an anti-His_6_ antibody.

We recently described an acetyl-lysyl-tRNA synthetase (AckRS)/tRNA_CUA_ pair that is derived from the *M. barkeri* (*Mb*) pyrrolysyl-tRNA synthetase/tRNA_CUA_ pair (Neumann et al., 2008). The AckRS/tRNA_CUA_ pair directs the incorporation of acetyl-lysine in response to the amber codon with high translational efficiency and fidelity to produce homogeneously acetylated protein. To improve the efficiency of this system further, we made a library that randomizes residues L266, A267, L270, Y271, and L274 in acetyl-lysyl-tRNA synthetase (Figure 1), and we selected for improved efficiency of acetyl-lysine incorporation as previously described (Neumann et al., 2008). These selections yielded an improved synthetase, which contains a single L266M mutation with respect to AcKRS-1. We named this synthetase AcKRS-3. Expression of myoglobin-his_6_ incorporating acetyl-lysine at position 4 from a myoglobin gene bearing an amber codon at position 4 demonstrates the improvement in protein expression using the AckRS-3/tRNA_CUA_ system.

**Figure 2:**
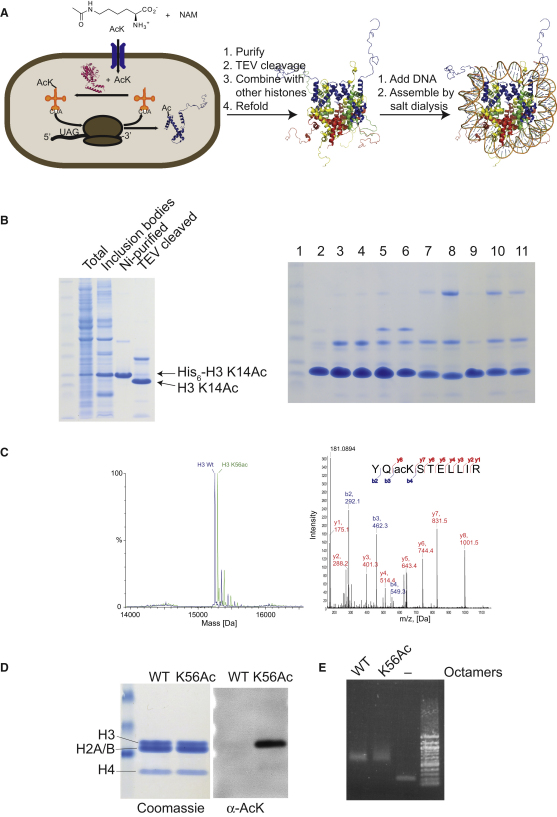
The Expression and Purification of Site-Specifically Acetylated Histones and the Assembly of Histone Octamers and Nucleosomes (A) Schematic illustration showing the recombinant expression of site-specifically acetylated recombinant histones in *E. coli* and their reconstitution into histone octamers and nucleosomes. (B) (Left) The expression, purification, and TEV cleavage of histone H3 K14Ac is followed by SDS PAGE. (Right) Purified and TEV cleaved site-specifically acetylated histones. (1) molecular weight marker, (2) H3 WT, (3) H3 K14Ac, (4) H3 K23Ac, (5) H3 K27Ac, (6) H3 K56Ac, (7) H2A WT, (8) H2A K9Ac, (9) H2B WT, (10) H2B K5Ac, and (11) H2B K20Ac. (C) Electrospray ionization mass spectrometry demonstrates that the protein is homogeneously acetylated, and MS/MS of tryptic peptides identifies the site of acetylation at lysine 56, as genetically encoded. The smaller peak to the right of the main peak is 98 Da heavier and corresponds to a phosphate from buffer associated with the histone. (D) H3 K56Ac assembles into octamers with comparable efficiency to unmodified H3. Denaturing (4%–12%) SDS-PAGE of assembled octamers. The acetylation of H3 in the octamer is confirmed by western blot with an anti-acetyl-lysine antibody. (E) Reconstitution of unmodified octamers and octamers bearing H3 K56Ac into nucleosomes with 197bp 601 DNA. Nucleosomes and free DNA were resolved by 0.8% agarose gel and stained with ethidium bromide.

In order to synthesize acetylated histone H3, we further created a vector that contains the *Mb*tRNA_CUA_ gene and an N-terminally hexahistidine-tagged histone H3 downstream of a T7 promoter (Figure S1 available online). We introduced an amber codon at position 56 and transformed this vector into BL21 *E. coli* bearing AcKRS-3. Cells were supplemented with 10 mM acetyl-lysine and 20 mM nicotinamide (to inhibit the *E. coli* deacetylase CobB) at OD 0.7, and protein expression was induced by addition of IPTG 30 min later. Like unmodified histone H3, the H3 K56Ac is overexpressed and found in inclusion bodies (Figure 2B). Histone H3 K56Ac was purified by denaturing Ni-NTA chromatography with a yield of 2 mg per liter of culture. Subsequent cleavage with TEV protease cleanly removed the N-terminal His_6_ tag. Electrospray ionization mass spectrometry (Figure 2C) demonstrates the homogeneous incorporation of a single acetyl-lysine residue, and MS/MS confirms that the amino acid is incorporated at the genetically encoded site. By simply moving the position of the amber codon in the H3 gene, we have made several other important acetylated variants of H3, including H3 K14Ac, K23Ac, and K27Ac. To further demonstrate the generality of the method, we have expressed and characterized other histones containing acetylated lysines at specific positions H2B K5, H2B K20, and H2A K9 (Figures 2B and Figure S2).

We assembled H3 K56Ac into histone octamers with H2A, H2B, and H4 using standard methods of refolding with a comparable efficiency to unmodified H3 (Luger et al., 1999), indicating that acetylation does not affect octamer formation in vitro (Figure 2D). We used these H3 K56Ac-containing histone octamers to assemble nucleosomes with DNA. The efficiency of octamers' formation with unacetylated H3 and H3 K56Ac was comparable (Figure 2E), again indicating that acetylation does not affect the efficiency of nucleosome formation.

### H3 K56Ac Does Not Affect Salt-Dependent Nucleosome Stability

The structure of the nucleosome core particle shows that H3 K56 is in an α helix that binds the last 10 bp of DNA at the entry/exit site, and H3 K56 itself makes a water-mediated contact between H3 K56 and the phosphate backbone of the DNA (Davey et al., 2002). Several groups have proposed that K56 acetylation affects the stability of the nucleosome or DNA breathing on the nucleosome and have suggested that this provides a structural, mechanistic, and energetic basis for observed cellular phenomena (Cosgrove et al., 2004; Hyland et al., 2005; Masumoto et al., 2005; Xu et al., 2005). However, there are no experimental data on the effect of this modification on nucleosome stability or DNA breathing. To investigate the effect of H3 K56 on nucleosome stability, we first compared the equilibrium stability as a function of NaCl concentration for nucleosomes containing H3 K56Ac and unacetylated H3 by fluorescence resonance energy transfer (FRET) using previously established assays and fluorophore positions (Li and Widom, 2004).

**Figure 3:**
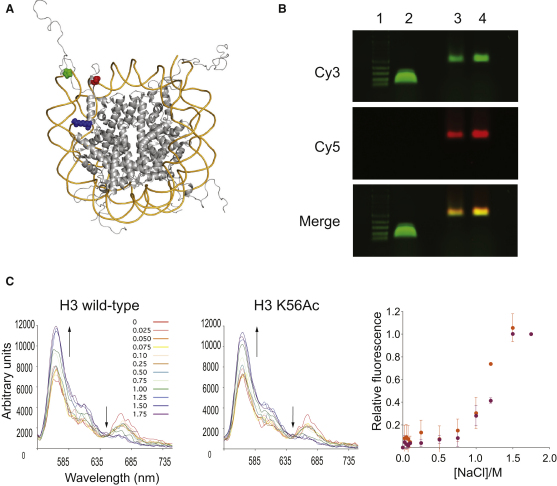
Nucleosome Stability and Dynamic Partial Unwrapping of Nucleosomal DNA Measured by FRET Using Three-Way Labeled Nucleosomes (A) Schematic of the nucleosome highlighting the locations of the fluorescence donor Cy3 (green) at the 5′ end of the DNA, the acceptor dye Cy5 (red) coupled to histone H2A K119C, and the site of acetylation, H3 K56Ac (blue). The figure was created using the PDB file 1KX5 and PyMOL (http://www.pymol.org). (B) Analysis of nucleosome reconstitution by 0.8% agarose gel electrophoresis, in which lane 1 = 100 bp DNA ladder, lane 2 = naked Cy3-labeled DNA, lane 3 = Cy5-labeled H2A K119C nucleosome reconstitution with wild-type H3, and lane 4 = Cy5-labeled H2A K119C nucleosome reconstitution with H3 K56Ac. (C) The salt-induced dissociation of nucleosome core particles can be monitored by FRET. (Left) Increasing [NaCl] from 0 (red) to 1.75 M (violet) leads to decreased FRET emission from Cy5 and increased Cy3 emission (arrows). Excitation wavelength was set at 515 nm. (Right) Equilibrium dissociation curves were obtained by monitoring changes in fluorescence donor and acceptor emission at 565 and 670 nm, respectively. Data were normalized using the upper and lower plateau values as baselines, with wild-type nucleosomes in orange and H3 K56Ac nucleosomes in magenta. The data represent the mean values, and the error bars represent ± 1 SD.

We placed a Cy3 FRET donor on the 5′ end of a 147 bp DNA nucleosome-positioning sequence (Lowary and Widom, 1998) and quantitatively labeled a K119C mutant of H2A with a Cy5 maleimide to create a FRET acceptor (Figure S3). We assembled nucleosomes with the Cy3-labeled DNA and octamers that contained the Cy5-labeled H2A and either H3 K56Ac or unacetylated H3 (Figures 3A and 3B). In each nucleosome, there are two Cy5 fluorescently labeled H2A molecules; however, only one of these H2A molecules is close enough to the Cy3 on the DNA to contribute significantly to FRET (Li and Widom, 2004). At high NaCl concentrations in which the nucleosome is dissociated, excitation of the Cy3 donor leads to strong emission centered on 565 nm, consistent with Cy3 fluorescence and negligible acceptor emission centered on 670 nm, as expected in the absence of FRET (Figure 3C). At low NaCl concentrations in which the nucleosome is intact, excitation of the Cy3 donor leads to a decreased donor emission at 565 nm and an increased Cy5 acceptor emission at 670 nm, consistent with a FRET signal. To assess the stability of H3 K56 acetylated nucleosomes, we followed the emission of donor and acceptor fluorophores as a function of [NaCl] for nucleosomes bearing H3 K56 acetylation and nucleosomes bearing unmodified H3 (Figure 3D). We found that acetylated and nonacetylated nucleosomes show comparable stability to NaCl through a range of concentrations that cover partial unwrapping of the DNA, dissociation of H2A/H2B dimers, and dissociation of H3/H4 dimers. These data indicate that acetylation of H3 K56 does not have a substantial effect on nucleosome stability, but the error in the assay does not allow us to distinguish small effects in partial unwrapping of the DNA that result from DNA breathing.

### H3 K56Ac Increases DNA Breathing in Mononucleosomes

To investigate the partial unwrapping of the DNA resulting from the spontaneous transient breathing of DNA from the histone core (Li and Widom, 2004), we used a recently developed combination of single-pair FRET (spFRET), alternating excitation (ALEX) selection, and native PAGE (Koopmans et al., 2009). Nucleosomes reconstituted on a nucleosome-positioning element DNA containing a Cy3B-ATTO647N FRET pair were separated from free DNA using native PAGE. The nucleosome-containing band was excised from the gel and imaged in a confocal microscope using rapidly alternating green and red excitation. Resulting photon bursts were separated into a green and a red channel. The fluorescence of each nucleosome that diffuses through the focus was analyzed for both FRET efficiency and fluorescent label stoichiometry. Finally, a distribution of FRET efficiencies was generated from individual nucleosomes that have both the donor and acceptor label. Using these stringent selections, we identified complexes that were folded into nucleosomes and contained both fluorescent labels. By examining this population, we were able to accurately assess the transient DNA conformations of individual nucleosomes.

**Figure 4:**
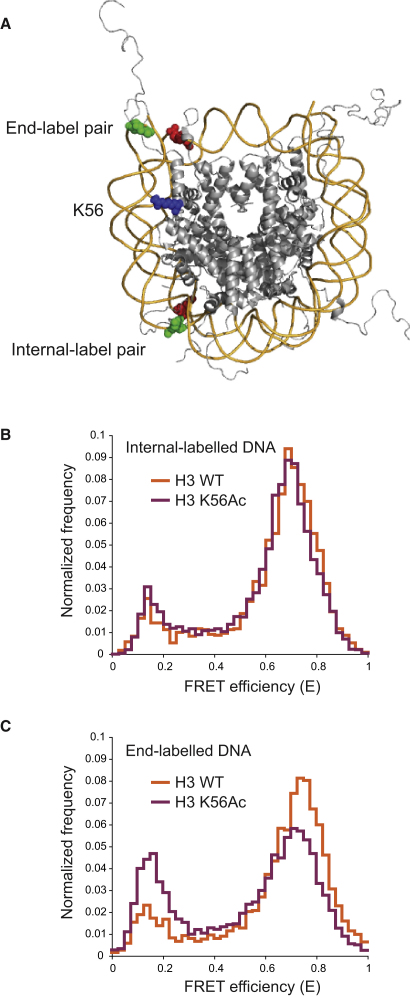
spFRET Experiments on Transient Unwrapping of DNA and DNA Breathing Demonstrate that K56 Acetylation Promotes Local Unwrapping Near the Entry-Exit Points of the Nucleosome (A) Schematic of the labeling positions on the nucleosome DNA. The end label fluorophore pair (Cy3B/Atto647N) is close to the entry-exit point of the nucleosome at position −2, and the internal label pair is at −27 from the entry-exit point. The position of K56 is shown in blue. The figure was created using the PDB file 1KX5 and PyMOL (http://www.pymol.org). (B and C) spFRET efficiency measured for nucleosomes reconstituted with internally or end-labeled DNA, respectively, using a combination of native PAGE, ALEX, and FCS as described in the Experimental Procedures.

To measure the effect of H3 K56 acetylation on DNA breathing, we used two label pairs. The first label pair was positioned 2 bp from the end of the DNA on the nucleosome and within 7 bp of the K56 location (end label, −2 position) and was located near the positions labeled in our bulk FRET experiments. The second label pair was positioned 27 bp from the end of the nucleosomal DNA and 20 bp internally to the K56-binding position (internal label, −27 position) (Figure 4A).

For the unmodified nucleosomes, 11% of the DNA is unwrapped (FRET efficiency < 0.3) at the internal position, and 13% of the DNA is unwrapped at the end position (Figures 4B and 4C) in the first turn of DNA. For H3 K56 acetylated nucleosomes, the fraction of nucleosomes in which the DNA is unwrapped at the end position is doubled to 28%, whereas the fraction of nucleosomes in which the DNA is unwrapped at internal position only increases 3%, from 11% to 14%. These data clearly indicate that H3 K56 acetylation is sufficient to cause a local increase in spontaneous DNA breathing at the entry exit point of the DNA on the nucleosome. This effect may increase the accessibility of nucleosomal DNA to other factors, such as nucleosome-remodeling complexes. Assuming that unwrapping of the end positions is required for unwrapping the internal pair, only 2% (13%–11%) of unacetylated nucleosome cores are unwrapped only at the end label position in the first turn of the DNA. In contrast, acetylated nucleosome cores are 14% (28%–14%) unwrapped only at the end label position. Comparison of the fraction of nucleosomes unwrapped only at the end label position suggests that acetylation increases DNA breathing within the last turn of DNA on the nucleosome core 7-fold.

### Formation of Higher-Order Chromatin Structure in Nucleosome Arrays Is Unaffected by H3 K56Ac

Compaction is a prerequisite for heterochromatin formation. Mutation of H3 K56 to a noncharged residue causes defects in silencing at telomeres (Hyland et al., 2005), in which K56 acetylation is normally less abundant (Xu et al., 2007). Moreover, failure to deacetylate K56 may lead to defective silencing at telomeres (Xu et al., 2007). These experiments suggest that H3 K56Ac may mediate, directly or indirectly, the compaction state of chromatin.

**Figure 5:**
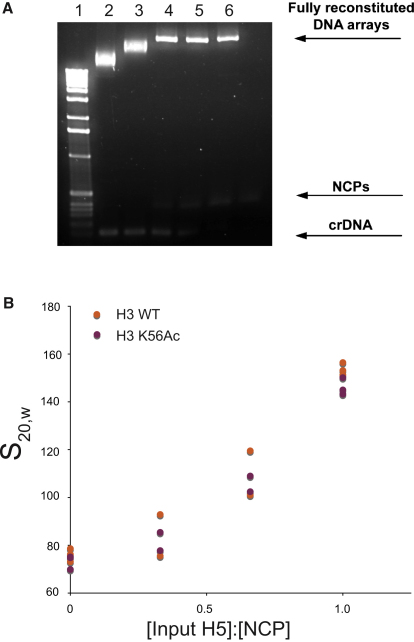
Assembly and Sedimentation Analysis of Nucleosome Arrays Bearing Homogeneously Acetylated Nucleosomes (A) Titration of purified histone H3 K56Ac octamers to assemble chromatin arrays containing 61 repeats of 197 bp of the 601 nucleosome-positioning DNA sequence. A retarded gel shift indicates loading of the DNA array with histone octamers. Excess histone octamer forms nucleosome core particles (NCPs) with competitor DNA (crDNA). Conditions of lane 4 were used to reconstitute DNA arrays in subsequent experiments. (B) DNA arrays were reconstituted with saturating amounts of histone octamer and with increasing amounts of H5 linker histone in order to induce compaction. Chromatin arrays were folded in 1 mM MgCl_2_ and 10 mM TEA (pH 7.4), and the degree of the compaction was measured quantitatively by sedimentation velocity analysis.

To investigate the direct effect of H3 K56Ac on chromatin compaction, we reconstituted nucleosome arrays. To do this, we assembled histone octamers containing H3 K56Ac or unmodified H3 on 61 repeats of the 197 base pair 601 nucleosome-positioning DNA sequence with increasing amounts of H5 linker histone (Figure 5A) (Huynh et al., 2005; Routh et al., 2008). These chromatin arrays were then folded in 1 mM MgCl_2_ and 10 mM TEA (pH 7.4). Sedimentation velocity analysis (Figure 5B) of the H3 K56Ac arrays reveals a compaction profile that is essentially indistinguishable from that of the arrays bearing wild-type H3. Our results suggest that H3 K56Ac has little effect on the compaction of the chromatin fiber and support the view that its effect on compaction suggested from in vivo experiments is either mediated by additional factors or is context dependent.

### The Effect of H3 K56Ac on Chromatin Remodeling

Chromatin immunoprecipitation (CHIP) experiments demonstrated a correlation between H3 K56Ac and SWI/SNF recruitment to activated promoters (Xu et al., 2005). Because SWI/SNF contains a bromodomain, we investigated the effect of K56 acetylation on the direct recruitment of SWI/SNF. We did not detect any difference in binding of K56 acetylated nucleosomes and nonacetylated nucleosomes to SWI/SNF (using electrophoretic mobility shift assays; data not shown), indicating that recruitment of SWI/SNF to K56-acetylated nucleosomes is either context dependent or is mediated by another factor. Similarly, we did not observe enhanced binding of the acetylated nucleosomes to RSC or Bdf1, which also contain bromodomains (data not shown). These experiments demonstrate that H3 K56Ac is not sufficient to directly recruit these remodelers.

**Figure 6:**
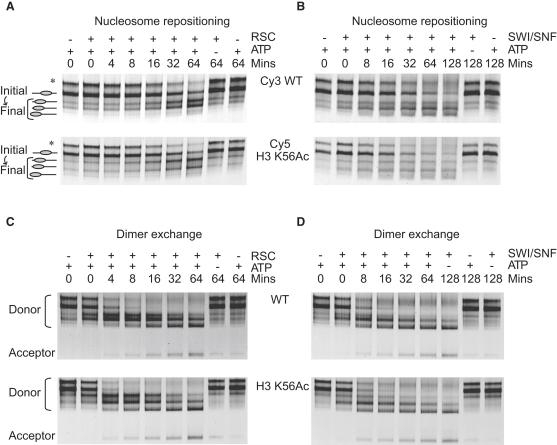
H3 K56 Acetylated Nucleosomes Cause Minimal Alteration to the Initial Rate of RSC or SWI/SNF Repositioning (A and B) Competitive repositioning assays were performed using 1 pmol each of H3 K56Ac and wild-type nucleosomes, 1 mM of ATP, and 41 fmol of RSC (A) or 115 fmol of SWI/SNF (B). A representative native PAGE gel of the repositioning assay is shown for each remodeler. The initial rate estimate for repositioning of H3 K56Ac nucleosomes relative to wild-type for RSC was 1.2 fold ± 0.1 (mean ± SE of the mean) and for SWI/SNF was 1.4 fold ± 0.2. Each experiment was repeated in triplicate. Asterisks indicate the P position. WT, wild-type. (C and D) H3 K56Ac and wild-type nucleosomes exhibit equivalent remodeler-driven dimer transfer. Remodeler dimer transfer was performed using 0.25 pmol of donor nucleosomes assembled with Cy5-labeled H2A onto 54A18 DNA fragments, 0.75 pmol of wild-type tetrasome acceptor assembled on 0W0 DNA fragments, 1 mM of ATP, and 83 fmol of RSC (C) or 230 fmol of SWI/SNF (D). For each dimer transfer experiment, a representative Cy5 scan of the native PAGE gel is shown. Both RSC and SWI/SNF caused a 1.2-fold increase of the percentage of dimer transfer for H3 K56Ac nucleosomes relative to wild-type at the finish of their respective time courses. As the SE of the mean was large in both cases, 0.1 and 0.2 for RSC and SWI/SNF, respectively, there was no significant change in the percentage of dimer transfer. Each experiment was repeated in triplicate.

It was proposed previously that H3 K56Ac modulates chromatin remodeling by SWI/SNF by facilitating access to the DNA at the entry-exit gate (Cosgrove et al., 2004). An H3 K56R mutant failed to recruit SWI/SNF as judged by ChIP analysis and failed to activate histone gene transcription (Xu et al., 2005). To investigate the effect of H3 K56Ac on remodeling by SWI/SNF and RSC, we compared the remodeling of nucleosomes containing H3 K56Ac and unmodified nucleosomes (Figures 6A and 6B). SWI/SNF repositioned H3 K56Ac-containing nucleosomes 1.4 fold ± 0.2 faster than unmodified control nucleosomes (Figure 6A), whereas RSC repositioned the acetylated nucleosomes 1.2 fold ± 0.1 faster than unmodified nucleosomes (Figure 6B). This observation is also consistent with the data obtained using H3 K56Q mutated nucleosomes, which were repositioned ∼1.3-fold faster than wild-type nucleosomes (Somers and Owen-Hughes, 2009). Collectively, these observations show that histone H3 K56 acetylation has a small effect (∼20%) on nucleosome redistribution by RSC and SWI/SNF. However, given that the effects of H3 K56Ac on in vivo transcriptional activation are 2- to 3-fold (Xu et al., 2005), it is possible that the modest increase in acetylation-dependent nucleosome repositioning contributes to the observed activation.

In addition to moving nucleosomes in *cis* along DNA, the Snf2 subfamily members have also been shown to displace H2A/H2B dimers from nucleosomes (Bash et al., 2006; Bruno et al., 2003). We therefore performed ATP-dependent H2A/H2B dimer transfer assays (Figures 6C and 6D) using H3 K56Ac nucleosomes to investigate whether this modification was able to influence this specific type of remodeling behavior. In brief, wild-type or H3 K56Ac nucleosomes were assembled with Cy5-labeled H2A onto Cy3-labeled 54A18 DNA fragments. A chromatin acceptor of wild-type H3/H4 tetramer assembled onto 0W0 DNA fragments was added at ∼3-fold molar excess. Following remodeling, the samples were resolved on a native PAGE gel, and the Cy5 histone dimer fluorescence was monitored. ATP-dependent dimer transfer for both RSC and SWI/SNF was not greatly affected by the H3 K56Ac donor nucleosomes relative to wild-type nucleosome controls (Figures 6C and 6D). Quantification of the data revealed that both RSC and SWI/SNF were no more efficient at H2A/H2B dimer transfer from H3 K56Ac nucleosomes than from unmodified nucleosomes, indicating that H3 K56Ac has a minimal effect on dimer transfer.

## Discussion

We have developed a method for the production of large quantities of histones bearing site-specifically defined and homogeneous acetylations. This method has allowed us to prepare histone H3 with 100% acetylation at K56 in the globular core of the histone. Using nucleosomes assembled with H3 K56Ac, we have measured the effect of H3 K56 acetylation on nucleosome stability and DNA breathing at the entry-exit points of the nucleosome. In summary, we find that, whereas H3 K56Ac does not affect the compaction of chromatin fibers, it affects the nucleosome core structure in a number of subtle ways: it increases DNA breathing at the entry-exit point of the nucleosome and has a small effect on remodeling of mononucleosomes. It is possible that these effects may be amplified within chromatin, leading to large-scale changes in accessibility and structure.

Understanding the effects of lysine acetylation on transcription, DNA replication, or DNA repair requires biochemical analysis of the effect of this modification on chromatin structure (Ruthenburg et al., 2007; Taverna et al., 2007a). Peptide models have allowed the interactions of histone tail modifications to be investigated (Bannister et al., 2001; Dhalluin et al., 1999; Jacobson et al., 2000; Kasten et al., 2004; Lachner et al., 2001; Li et al., 2006; Wysocka et al., 2006), but these experiments cannot address the direct or indirect effects of modifications on nucleosome structure, the higher-order structure of chromatin, or the effect of modifications on the interaction with other factors and remodelers within the context of intact chromatin. Moreover, peptide models cannot be used to probe the role of the emerging modifications in the globular core of histone proteins, the most prominent of which is, perhaps, H3 K56 acetylation. From the outset, H3 K56 acetylation was hypothesized to strongly destabilize this DNA-histone interaction at the entry-exit point (Hyland et al., 2005; Masumoto et al., 2005; Xu et al., 2005, 2007). This was assumed to affect the structure of chromatin, especially during compaction (Hyland et al., 2005; Xu et al., 2007), and has been implicated in silencing at telomeres (Xu et al., 2007). The acetylation might directly affect compaction or act to recruit factors that affect the remodeling and compaction of chromatin. Our data demonstrate that H3 K56Ac is not sufficient to cause the 2- to 3-fold changes in compaction observed for H4 K16 (Robinson et al., 2008; Shogren-Knaak et al., 2006). The effect of H3 K56 acetylation on silencing is, therefore, more subtle and must depend on the simultaneous presence of other modifications or on the modification-dependent recruitment or action of other factors.

Single-molecule FRET experiments on acetylated nucleosomes, which are possible with the homogeneous material produced by our method, demonstrate that H3 K56 acetylation is sufficient to cause a 7-fold increase in DNA breathing on the nucleosome. These results are consistent with the observed increase in MNase sensitivity of yeast chromatin bearing an H3 K56Q mutation (Masumoto et al., 2005). Our data also support the proposal that deacetylation of H3 K56Ac by Sir2 acts to close the entry-exit gates of DNA around the histone octamer (Xu et al., 2007). Furthermore, the 7-fold increase in DNA breathing observed in our single-molecule experiments may well explain a number of phenotypes reported for mutations in H3 K56 or the enzymes involved in its modification. For example, the Grunstein lab reported (using ChIP assays) that wild-type yeast recruited the remodeler SWI/SNF 2- to 3-fold more efficiently to the HTA1 promoter than an H3 K56R mutant (Xu et al., 2005). Changes seen in gene expression levels caused by deletion of Spt10 (an enzyme required for the acetylation of H3 K56 on histone genes) are of the same magnitude and overlap with the changes seen for mutations in H3 K56 (Xu et al., 2005). H3 K56Ac is also involved in the activation of the PHO5 promoter (Williams et al., 2008). The loss of nucleosomes from the PHO5 promoter is slowed ∼2 fold by a H3 K56R mutation. H3 K56Q mutants also affect telomeric chromatin structure, leading to a 4- to 6-fold increase in the mRNA levels of a telomere proximal gene (Xu et al., 2007). The magnitude of the physical changes resulting from H3 K56Ac are of the same order of magnitude as the increases in, for instance, transcription, suggesting that the biological processes are directly regulated by the enhanced nucleosome plasticity.

The method that we present for genetically encoding acetyllysine will facilitate investigation into the roles of histone acetylation within the same nucleosome, as well as the roles of multiple acetylations on a single histone (Wang et al., 2007). We have recently extended our method for genetically encoding methylation (Nguyen et al., 2009). Our method of acetylation is compatible with native chemical ligation (Dawson et al., 1994; Ferreira et al., 2007; McGinty et al., 2008; Shogren-Knaak et al., 2006), as well as methods for installing methylation analogs (Simon et al., 2007), and it will therefore be possible to introduce acetylation in combination with other histone modifications, such as ubiquitylation and methylation on individual histones and nucleosomes. Combining the full arsenal of methods, synthetic and genetic, for installing histone posttranslational modifications in chromatin will be increasingly important for defining the combinatorial role of modifications (Ruthenburg et al., 2007; Taverna et al., 2007b) and providing a biochemical understanding of biological phenomena.

## Experimental Procedures

Information on library design and selection, mass spectrometric analysis of recombinant histones, and the purification of remodeling complexes can be found in the Supplemental Data. Standard methods and assays, such as the reconstitution of histone octamers and nucleosome arrays and their analysis by sedimentation velocity, bulk FRET, and remodeling assays, can also be found there.

### Expression and Purification of Acetylated Histones H2A, H2B, and H3

BL21 DE3 (for H3) or Rosetta DE3 (for H2A and H2B) cells were transformed with plasmid pAcKRS-3 and pCDF PylT-1 carrying the ORF for the histone with amber codons at the desired sites. The cells were grown overnight in LB supplemented with 50 μg/ml kanamycin and 50 μg/ml spectinomycin (LB-KS). One liter of prewarmed LB-KS was inoculated with 50 ml overnight culture and was incubated at 37°C. At OD600 of 0.7–0.8, the culture was supplemented with 20 mM nicotinamide (NAM) and 10 mM acetyl-lysine (AcK). Protein expression was induced 30 min later by addition of 0.5 mM IPTG. Incubation was continued at 37°C, and cells were harvested 3–3.5 hr after induction, washed with PBS 20 mM NAM, and stored at −20°C.

The pellet was resuspended in 30 ml PBS supplemented with 20 mM NAM, 1 mM PMSF, 1 × PIC (Roche), 1 mM DTT, 0.2 mg/ml lysozyme, and 0.05 mg/ml DNase I and was incubated for 20 min with shaking at 37°C. Cells were lysed by sonication (output level 4 for 2 min on ice). Extracts were clarified by centrifugation (15 min, 18,000 rpm, SS34), and the pellet was resuspended in PBS supplemented with 1% Triton X-100, 20 mM NAM, and 1 mM DTT. The samples were centrifuged as above and washed again, once with the same buffer and then twice with the same buffer without Triton X-100. The pellet was macerated in 1 ml DMSO and incubated for 30 min at room temperature. Twenty-five milliliters of 6 M guanidinium chloride, 20 mM Tris, and 2 mM DTT (pH 8.0) were used to extract the histone proteins from the pellet. The samples were incubated for 1 hr at 37°C with shaking, centrifuged as above, and loaded onto a pre-equilibrated Ni^2+^-NTA column (QIAGEN). The column was washed with 100 ml 8 M urea, 100 mM NaH_2_PO_4_, and 1 mM DTT (pH 6.2). Bound proteins were eluted with 7 M urea, 20 mM sodium acetate, 200 mM NaCl, and 1 mM DTT (pH 4.5).

The eluates containing the protein were combined and dialysed at 4°C against 5 mM β-mercaptoethanol (two times against the 100-fold volume). The solution was made up of 50 mM Tris/HCl (pH 7.4) and supplemented 1:50 with 4 mg/ml TEV. The reaction was incubated for 5 hr at 30°C. Afterward, salts were removed by dialysis as above, and the protein was lyophilized.

### Single-Molecule FRET

Mononucleosomes were reconstituted on a fluorescently labeled 155 bp DNA template containing a 601 nucleosome-positioning sequence as described (Koopmans et al., 2007). In brief, the template DNA was prepared by PCR and was labeled with Cy3B (donor) and ATTO647N (acceptor) by incorporation of fluorescently labeled, HPLC-purified primers (IBA GmbH, Göttingen, Germany). Nucleosome reconstitutions were analyzed with 5% native poly-acrylamide gel electrophoresis (PAGE). A sample of 0.1–1 pmol was loaded on the gel (29:1 bis:acrylamide, 0.2× TB). The gel was run at 19 V/cm at 4°C for 80 min and visualized with a gel imager (Typhoon 9400, GE, Waukesha, WI, USA). The band corresponding to reconstituted nucleosomes was excised and put on a home-built confocal microscope equipped with a 60× water immersion microscope objective (NA = 1.2, Olympus, Zoeterwoude, The Netherlands) and two single-photon avalanche photodiodes (SPCM AQR-14, Perkin-Elmer [EG&G], Waltham, MA, USA). The photodiodes were read out with a TimeHarp 200 photon counting board (Picoquant GmbH, Berlin, Germany). A 515 nm diode-pumped solid-state laser (Cobolt, Solna, Sweden) and a 636 nm diode laser (Power Technology, Little Rock, AR, USA) were alternated at 20 kHz for excitation. In a typical experiment, data were collected for 10 min, and 1000–5000 bursts of fluorescence were detected.

Photon arrival times in the donor and acceptor channel were sorted according to excitation period, resulting in four photon streams: I_515_^D^, donor emission during green excitation; I_515_^A^, acceptor emission during green excitation; I_636_^D^, donor emission during red excitation; and I_636_^A^, acceptor emission during red excitation. The total fluorescence emission was analyzed with a burst detection scheme as described (Eggeling et al., 2001). A burst was selected if a minimum of 100 photons arrived subsequently, with a maximum interphoton time of 100 μs. For each burst, we calculated the apparent FRET efficiency E (also known as proximity ratio): $E={\text{N}}_{\text{515}}^{\text{A}}/{\text{N}}_{\text{515}}^{\text{A}}+\gamma {\text{N}}_{\text{515}}^{\text{D}}$ and the apparent label stoichiometry S: $E={\text{N}}_{\text{515}}^{\text{A}}+\gamma {\text{N}}_{\text{515}}^{\text{D}}/{\text{N}}_{\text{515}}^{\text{A}}+\gamma {\text{N}}_{\text{515}}^{\text{D}}+{\text{N}}_{\text{636}}^{\text{A}}$, in which N_515_^D^, N_515_^A^, and N_636_^A^ are number of photons in the burst from the different photon streams, and γ is a parameter to correct for photophysical properties of the dyes, in our case equal to unity. The excitation powers were chosen such that N_515_^A^ + g N_515_^D^ ≈N_636_^A^, resulting in S∼0.5 for doubly labeled nucleosomes. Only nucleosomes with 0.2 < S < 0.8 were selected for FRET analysis.

## Supplemental Data

Document S1. Supplemental Experimental Procedures and Three Figures
