# Supplementary material for: A Method for Genetically Installing Site-Specific Acetylation in Recombinant Histones Defines the Effects of H3 K56 Acetylation
Source: Mol Cell. 2009 Oct 9;36(1):153–63. doi: 10.1016/j.molcel.2009.07.027 (PMC2856916; doi:10.1016/j.molcel.2009.07.027)
Supplement: Document S1. Supplemental Experimental Procedures and Three Figures [file mmc1.pdf]

Molecular Cell, *Volume 36*

Supplemental Data

A Method for Genetically Installing Site-Specific Acetylation in Recombinant Histones Defines the Effects of H3 K56 Acetylation

Heinz Neumann, Susan M. Hancock, Ruth Buning, Andrew Routh, Lynda Chapman, Joanna Somers, Tom Owen-Hughes, John van Noort, Daniela Rhodes, and Jason W. Chin

Supplemental Figures

A

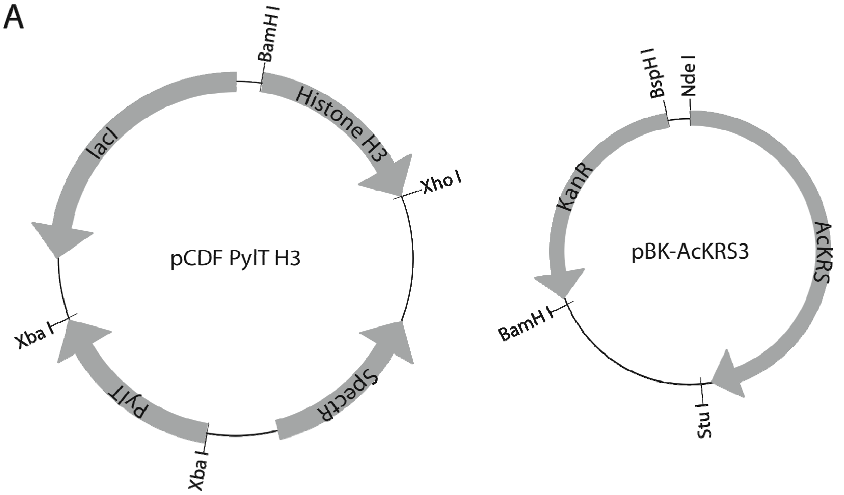

B

H2A

GSGRGKQGGKTRAKAKTRSSRAGLQFPVGRVHRLLRKGNYAERVGAGAPV
YLAAVLEYLTAEILELAGNAARDNKKTRIIPRHLQLAVRNDEELNKLLGR
VTIAQGGVLPNIQSVLLPKKTESSKSAKSK

H2B

GAKSAPAPKKGSKKAVTKTQKKDGKKRRKTRKESYAIYVYKVLKQVHPDT
GISSKAMSIMNSFVNDVFERIAGEASRLAHYNKRSTITSREIQTAVRLLL
PGELAKHAVSEGTKAVTKYTSAK

H3

GRTKQTARKSTGGKAPRKQLATKAARKSAPATGGVKKPHRYRPGTVALRE
IRRYQKSTELLIRKLPFQRLVREIAQDFKTDLRFQSSAVMALQEASEAYL
VALFEDTNLCAIHAKRVTIMPKDIQLARRIRGERA

Supplementary Figure 1: A. The two-plasmid system used for histone expression. B. Amino acid sequence of the histones produced from the pCDF PylT plasmids after TEV cleavage.

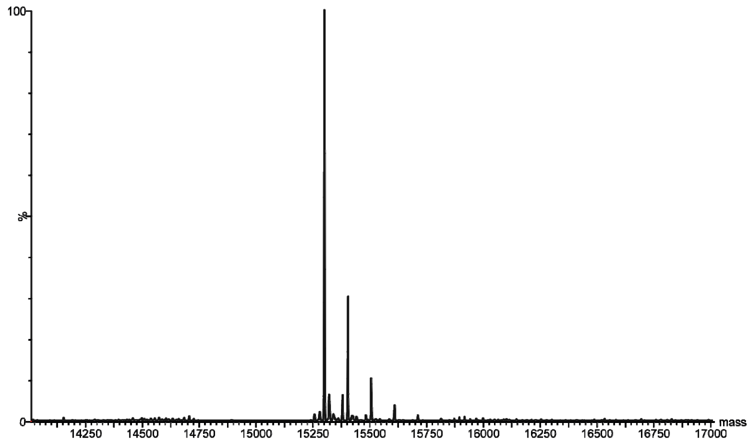

Supplementary Figure 2A. Molecular mass of H3 K14Ac confirmed by electrospray ionization mass spectrometry. His_6-tagged Histone H3 K14ac was expressed in *E. coli* BL21 DE3, purified by Ni^{2+} chromatography and cleaved with TEV protease. The observed mass of the protein (15299.0 Da) corresponds well to the theoretical mass of a singly acetylated histone H3 (15298.9 Da). Additional peaks of higher mass result from non-covalent phosphate adducts.

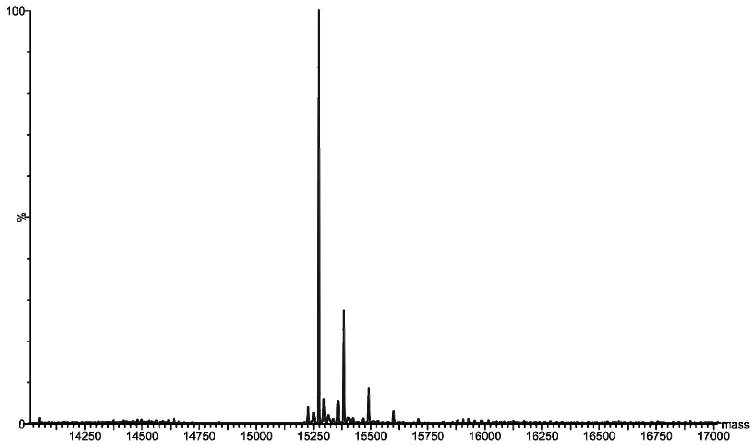

Supplementary Figure 2B. Molecular mass of H3 K23Ac confirmed by electrospray ionization mass spectrometry. His_6-tagged Histone H3 K23Ac was expressed in *E. coli* BL21 DE3, purified by Ni^{2+} chromatography and cleaved with TEV protease. The observed mass of the protein (15299.5 Da) corresponds well to the theoretical mass of a singly acetylated histone H3 (15298.9 Da). Additional peaks of higher mass result from non-covalent phosphate adducts.

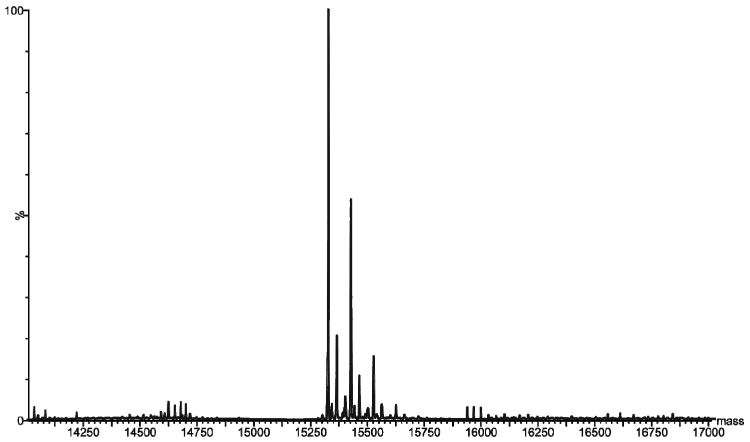

Supplementary Figure 2C. Molecular mass of H3 K27Ac confirmed by electrospray ionization mass spectrometry. His_6-tagged histone H3 K27ac was expressed in *E. coli* BL21 DE3, purified by Ni^{2+} chromatography and cleaved with TEV protease. The observed mass of the protein (15297.4 Da) corresponds well to the theoretical mass of a singly acetylated histone H3 (15298.9 Da). Additional peaks of higher mass result from non-covalent phosphate adducts.

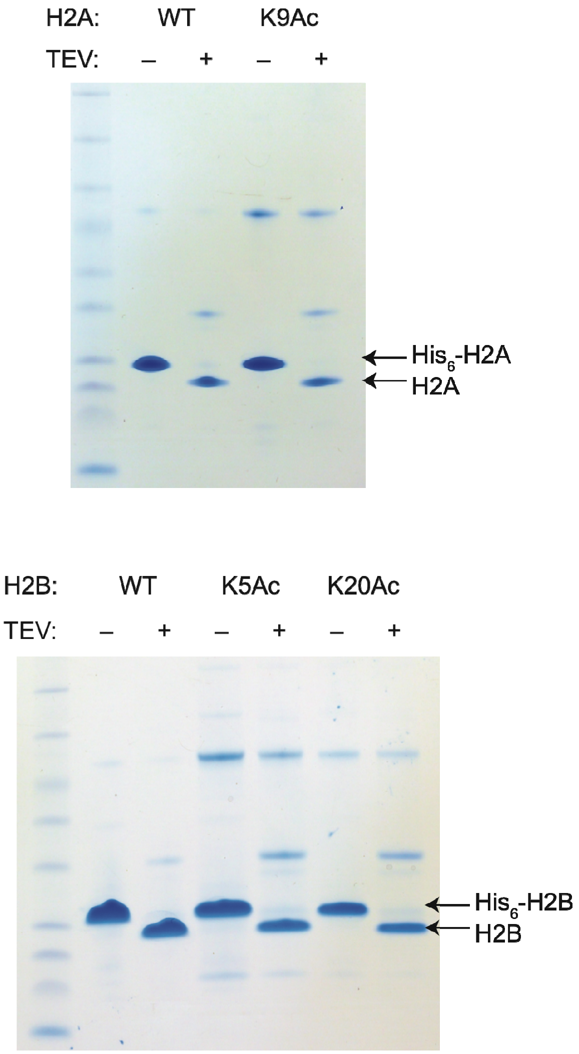

Supplementary Figure 2D: Purification and TEV protease cleavage of histones H2A and H2B. Proteins were expressed in *E. coli* Rosetta DE3 and purified by Ni^{2+} chromatography. The proteins were subsequently cleaved with TEV protease (0.08 mg/ml) and analysed on 4-12% SDS-PAGE gels. Samples of each protein are shown before and after TEV cleavage.

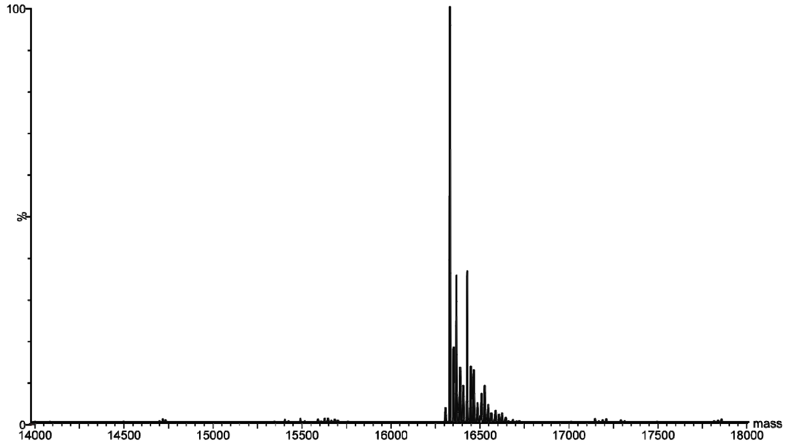

Supplementary Figure 2E. Molecular mass of H2A K9Ac confirmed by electrospray ionization mass spectrometry. His_6-tagged histone H2A K9Ac was expressed in *E. coli* Rosetta DE3, purified by Ni^{2+} chromatography and cleaved with TEV protease. The observed mass of the protein (16327.0 Da) corresponds well to the theoretical mass of a singly acetylated histone H2A lacking the N-terminal methionine (16325 Da).

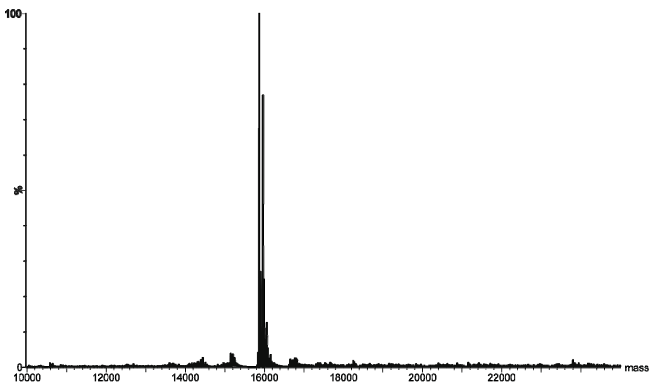

Supplementary Figure 2F. Molecular mass of H2B K5Ac confirmed by electrospray ionization mass spectrometry. His_6-tagged histone H2B K5Ac was expressed in *E. coli* Rosetta DE3, purified by Ni^{2+} chromatography and cleaved with TEV protease. The observed mass of the protein (15870.0 Da) corresponds well to the theoretical mass of a singly acetylated histone H2B lacking the N-terminal methionine (15869 Da). Additional peaks of higher mass mainly result from non-covalent phosphate adducts.

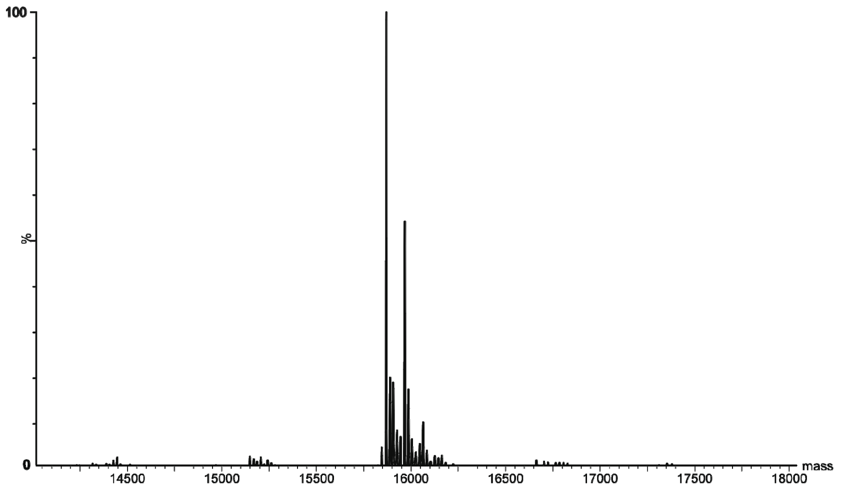

Supplementary Figure 2G. Molecular mass of H2B K20Ac confirmed by electrospray ionization mass spectrometry. His_6-tagged histone H2B K20Ac was expressed in *E. coli* Rosetta DE3, purified by Ni^{2+} chromatography and cleaved with TEV protease. The observed mass of the protein (15871.0 Da) corresponds well to the theoretical mass of a singly acetylated histone H2B lacking the N-terminal methionine (15869 Da). Additional peaks of higher mass mainly result from non-covalent phosphate adducts.

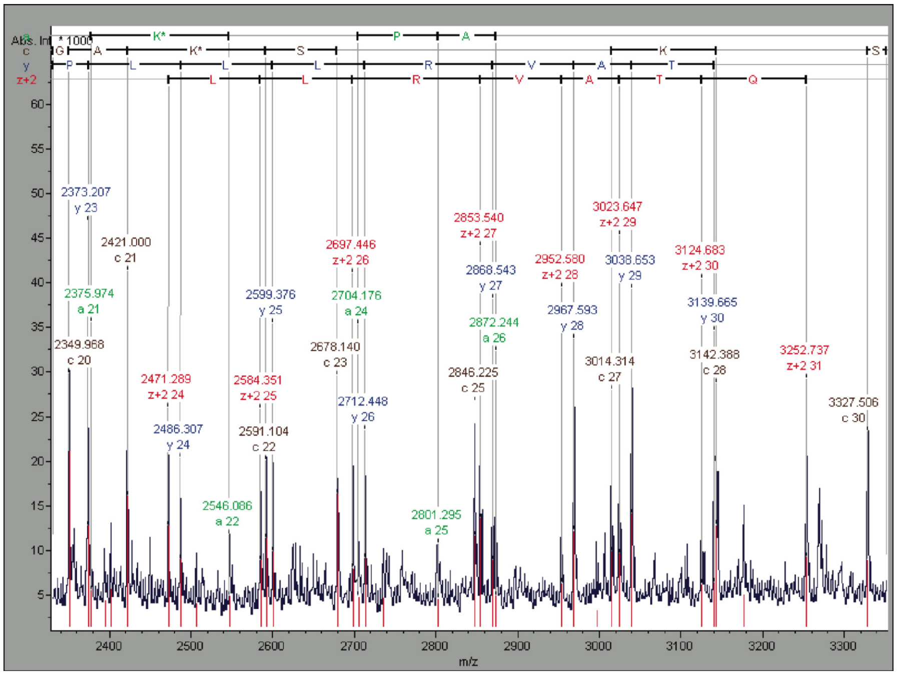

Supplementary Figure 2H: Top down sequencing of histone H2A K9Ac confirms site-specific incorporation of acetyl-lysine. The purified protein was subjected to MALDI top down sequencing as described in the Materials and Methods. The protein sequence inferred from the mass differences of individual ions is indicated above the spectrum and confirms the site-specific incorporation of acetyl-lysine.

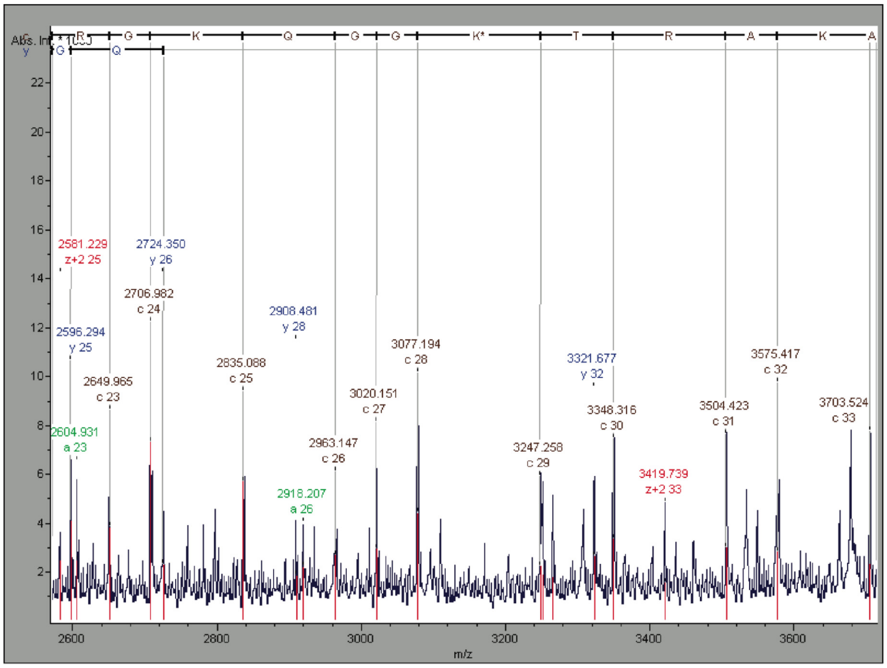

Supplementary Figure 2I: Top down sequencing of Histone H2B K5Ac confirms site-specific incorporation of acetyl-lysine. The purified protein was subjected to MALDI top down sequencing as described in the Materials and Methods. The protein sequence inferred from the mass differences of individual ions is indicated above the spectrum and confirms the site-specific incorporation of acetyl-lysine.

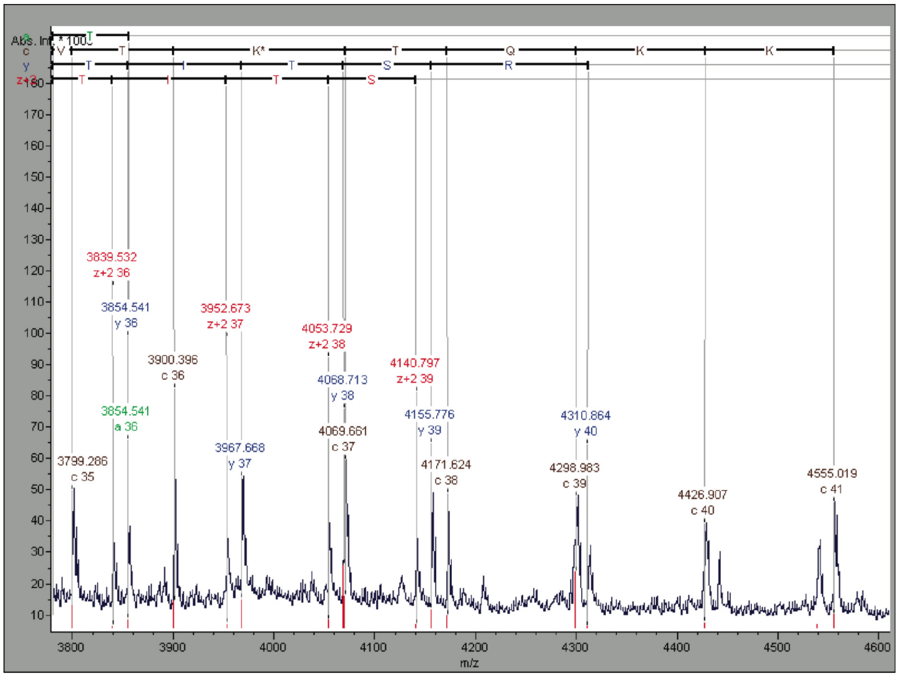

Supplementary Figure 2J: Top down sequencing of Histone H2B K20Ac confirms site-specific incorporation of acetyl-lysine. The purified protein was subjected to MALDI top down sequencing as described in the Materials and Methods. The protein sequence inferred from the mass differences of individual ions is indicated above the spectrum and confirms the site-specific incorporation of acetyl-lysine.

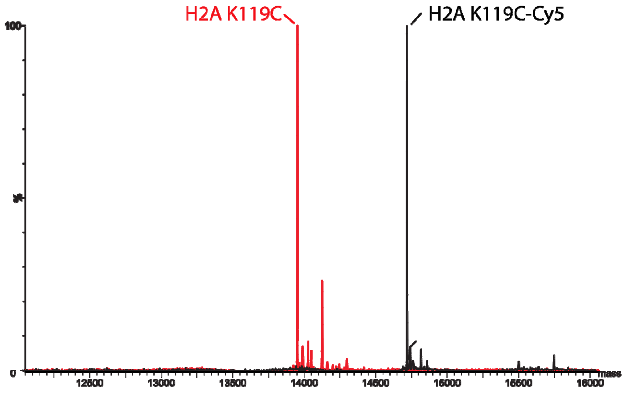

Supplementary Figure 3. Molecular mass of H2A K119C and its labelling with maleimide-Cy5 confirmed by electrospray ionization mass spectrometry. Histone H2A K119C was expressed in *E. coli* Rosetta DE3 pLysS, purified as described (Luger et al. 1999) and modified with maleimide-Cy5 (GE Healthcare). The unmodified (red) and the Cy5 labelled (black) proteins were analysed by ESI mass spectrometry. The main peak for the unmodified protein (13925.6 Da) corresponds well to the theoretical mass of histone H2A lacking the N-terminal methionine (13925.1 Da). The labelled histone gave rise to a peak of 14705.2 Da, which corresponds well to the calculated mass of 14703.1 Da. The spectra demonstrate a virtually complete labelling of the histone.

Supplemental Experimental Procedures

Library design and selection

The kanamycin resistance gene on plasmid pBK-JYRS was replaced by cloning an ampicillin resistance cassette (amplified by PCR from pJC72 with primers 5'-tgg tca tga tac att caa ata tgt atc cgc tc-3' and 5'-cga gga tcc tct gac gct cag tgg aac gaa aac-3') into the restriction sites BspHI and BamHI. Subsequently, pBK-AcKRS1amp was created by replacing the open reading frame of MjYRS with the NdeI/StuI fragment from plasmid pBK-AcKRS1 containing the ORF of AcKRS1. This plasmid was then used as a template in the generation of a library of PylS mutants. A single round of inverse PCR (Rackham and Chin, 2005) (with primers 5'-gcg cag gtc tca ccg atg DTK NNK ccg acc DTK HWK aac tat NYK cgt aaa ctg gat cgt att ctg ccg ggt c-3' and 5'-gcg cag agt agg tct cat cgg acg cag gca cag gtt ttt atc cac gcg gaa aat ttg-3') was performed to partially randomize codons for L266 and L270 (to F, L, I, M and V), Y271 (to F, L, I, M, Y, H, Q, N and K) and L274 (to F, L, I, M, V, S, P, T, A). The codon for A267 was mutated to decode all 20 natural amino acids in this library. The PCR product was first digested with DpnI and BsaI and then re-circularized by ligation. Transformation of electro-competent DH10B with the ligation produced 10^8 transformants, covering the theoretical diversity of the library (2.2×10^5) by more than 99.99%. Selection of mutants specific for acetyl-lysine was carried out as described. Eventually, the ORF of AcKRS-1 in the original pBKAcKRS-1 plasmid was replaced with AcKRS-3 using the restriction sites NdeI and StuI.

Mass spectrometry

A 3 µM solution of H3 K56ac in 100 mM $(NH_4)HCO_3$ was digested with trypsin overnight. An aliquot of this digest was separated by nanoscale liquid chromatography (LC Packings) on a reversed-phase C18 column (150 × 0.075 mm internal diameter, flow rate 0.25 µl min^{-1}). The eluate was introduced directly into a Q-STAR pulsar i hybrid tandem mass spectrometer (MDS Sciex). The spectra were searched against a NCBI non-redundant database with MASCOT MS/MS Ions search (www.matrixscience.com). The doubly charged ion with m/z 646.88 matched to the acetylated peptide. The acetylation site was confirmed by manual inspection of the fragmentation series.

Protein total mass was determined on an LCT time-of-flight mass spectrometer with electrospray ionization (ESI). (Micromass). Proteins were rebuffered to 20 mM $(NH_4)HCO_3$ pH 7.5 and diluted 1:100 into 50% methanol, 1% formic acid. Samples were infused into the ESI source at 10 ml min^{-1}, using a Harvard Model 22 infusion pump (Harvard Apparatus) and calibration performed in positive ion mode using horse heart myoglobin. 60–80 scans were acquired and added to yield the mass spectra. Molecular masses were obtained by deconvoluting multiply charged protein mass spectra using MassLynx version 4.1 (Micromass). Theoretical molecular masses of wild-type proteins were calculated using Protparam (http://us.expasy.org/tools/protparam.html), and theoretical masses for unnatural amino acid containing proteins adjusted manually. Where indicated protein total mass and acetylation position sequencing was performed using a top down approach, in these cases in-source decay (ISD) spectra were acquired in reflectron mode on an Ultraflex III TOF/TOF mass spectrometer (Bruker Daltonics, Bremen, Germany) using a 2,5-dihydroxy benzoic acid matrix.

Histone octamer reconstitution

Lyophilized histones were dissolved at an equivalent of 1 mg H2A per ml in unfolding buffer (7 M guanidinium chloride in 20 mM Tris, pH 7.4, 10 mM DTT) and mixed in stoichiometric amounts (Luger et al., 1999). A 2 ml reaction was incubated for 3 h at room temperature with gentle agitation and dialysed against three times 250 ml refolding buffer (2 M NaCl, 10 mM Tris pH 7.4, 1 mM EDTA, 5 mM β-mercaptoethanol) at 4ºC. Precipitates were removed by centrifugation (5 min, 25000 g, 2ºC) and filtered using a SpinX column. Octamers were then separated by gel filtration using a Superdex200 column equilibrated with refolding buffer.

Labelling of H2A K119C with maleimide-Cy5

The K119C mutation was introduced into pET3 H2A by Quikchange and H2A K119C was expressed and purified following published procedures (Luger et al., 1999). The protein was rebuffered to degassed PBS containing 1 mM TCEP using a PD10 column. In a 1 ml reaction 2 mg of the protein were reacted with 400 µg maleimide-Cy5 for 18 h at 4ºC. The reaction was then dialysed against two times 500 ml 5 mM β-mercaptoethanol over night at 4ºC and lyophilized. Analysis by ESI-TOF MS showed that the reaction had gone to completion (See supplementary

information).

Measuring equilibrium stability of nucleosomes by FRET

Using the high-affinity nucleosome positioning sequence 601 (Lowary and Widom, 1998) as a template, the 147 bp dominant nucleosome position (Dorigo et al., 2003) on the 282 bp sequence 601 was generated with the following primers: Cy3-LE19: 5'-(Cy3-C)TG GAG AAT CCC GGT GCC G-3', RE23: 5'-ACA GGA TGT ATA TAT CTG ACA CG-3' to produce Cy3-labelled 147 bp 601 DNA. The PCR was followed by agarose gel until the oligonucleotide primers were exhausted.

Histone octamers containing unacetylated H3 or H3 K56Ac and Cy5-labelled H2A were reconstituted as described above. Octamers and 147 bp Cy3-DNA were mixed in high-salt buffer (2 M NaCl, 10 mM Tris–HCl (pH 7.4), 1 mM EDTA, 5 mM β-mercaptoethanol) and nucleosome core particles assembled by a continuous dialysis method in which the NaCl concentration was reduced from 2.0 M to 10 mM over a 16 hour period at 4°C. The stoichiometry of histone octamer binding was assessed by gel mobility-shift assays in 0.8% (w/v) agarose gels imaged with a Typhoon Imager.

Fluorescence experiments were carried out at room temperature (~23°C) on a Tecan safire^2 spectrophotometer. Nucleosome samples were excited at 515 nm and emission spectra were collected from 535-750 nm. Emission wavelength maxima were observed at 565 nm for Cy3 and 670 nm for Cy5. Samples were incubated for at least 5 minutes at each salt concentration prior to each reading, as it has been previously demonstrated that longer incubation does not lead to any further change in emission intensity (Park et al., 2004), indicating that an equilibrium has been achieved within 5 min. All samples contained a final concentration of ~8 nM nucleosome core particles. Relative fluorescence intensity was calculated from FRET donor intensity/ FRET acceptor intensity and data were normalized using the upper and lower plateau values as baselines.

Production of DNA arrays for compaction

To produce and purify the DNA arrays, *E. coli* DH5α containing a pUC18 vector with the DNA array insert was grown overnight in 1 L of 2×TY (37°C, 210 rpm). For blunt-ended release, multimer arrays (12 kbp) were excised by digestion with EcoRV. The vector was digested into smaller products (<1 kbp) using HaeII and DraI. The

array DNA was separated from the fragments by selective polyethylene glycol (PEG) precipitation of long DNA fragments using 5-8% PEG 6000 in 0.5 M NaCl. The purified array DNA was phenol/chloroform extracted, ethanol precipitated, and the DNA pellets were re-suspended in 2 M NaCl, 10 mM TEA and 1 mM EDTA.

Competitor DNA (crDNA) was obtained from chicken erythrocyte nuclei. Mono-nucleosomes with approximately 147 bp of mixed sequence DNA were obtained by limited micrococcal nuclease digest of long chicken chromatin. Phenol/chloroform extraction removed bound histones.

Reconstitution of nucleosome arrays

Nucleosome arrays were reconstituted at 25 μg/ml DNA using our *in vitro* reconstitution method (Huynh et al., 2005). The molar input ratio of histone octamer required to obtain saturation was empirically determined. For compaction studies, the linker histone (H5) was added to the reconstitution in increasing concentrations. Mixed sequence crDNA (~147 bp) was added in all reconstitutions at a crDNA:601 DNA array mass ratio of 1:2 to prevent super-saturation of the 601 DNA arrays with excess histone octamer, ensuring that one histone octamer was bound per 601 DNA repeat. Following reconstitution, chromatin arrays were dialysed into folding buffer containing 1 mM $MgCl_2$ and 10 mM TEA pH 7.4. The recombinant chromatin arrays — whether acetylated or not — precipitated readily at higher divalent cation concentration, regardless of the concentration of monovalent ion used. The reconstitution and folding of nucleosome arrays was monitored by electrophoresis in native agarose gels.

Sedimentation velocity analysis data

Sedimentation velocity analysis data were obtained using a Beckman XL-A analytical ultracentrifuge equipped with scanner optics. Optical density was measured at 260 nm with an initial absorbance between 0.5 and 1.2. Sedimentation analysis was carried out for 2 h at 5°C at speeds between 18,000 r.p.m. in 12 mm double-sector cells and a Beckman AN60 rotor. Prior to analysis, samples and blanking buffer were placed in cells to settle for approximately one hour as this dramatically improved reproducibility of results. Sedimentation coefficients were determined using the time-derivative method described by Stafford (Stafford, 1992), using John Philo's Dcdt+

data analysis program (version 2.05) (Philo, 2006). Sedimentation coefficients were corrected to $S_{20,w}$. Partial specific volumes were calculated for all sample assuming values of 0.725 and 0.55 for protein content and DNA content respectively. Partial specific volumes are thus adjusted to account for linker histone content. Solvent viscosity and solvent density were corrected according to buffer composition.

Purification of remodeling complexes

Yeast strains TAP tagged for RSC (Saha et al., 2002) and SWI/SNF (Chandy et al., 2006) were purified as described previously (Ferreira et al., 2007). The SWI/SNF used for testing H3 K56 acetylated nucleosomes was a kind gift from Salma Mahmood and was purified essentially as described (Ferreira et al., 2007) but with the following changes: 6 L of cells were grown in 1×yeast extract, peptone, adenine, D-glucose. The cells were disrupted using 0.5 mm glass beads in a Bead Beater (Biospec Products Incorporated) using 10 pulses of 30 s ON, 1 min OFF. SWI/SNF wash and storage buffers contained 150 mM NaCl.

Mononucleosome repositioning assays

Nucleosomes were assembled onto DNA fragments described with the nomenclature *aBc*, with *a* and *c* are numbers that describe the length of the upstream and downstream bp extensions, respectively. *B* is the nucleosome positioning sequence source, with A and W representing the mouse mammary tumor virus (MMTV) nucleosome A (Flaus and Richmond, 1998) and 601.3 sequence (Anderson et al., 2002), respectively. Fluorescently labelled oligos were from Eurogentec (Belgium) and unlabelled oligos from the Oligonucleotide Synthesis Laboratory (University of Dundee, UK). The oligo sequences to amplify the 54A18 fragment are 5'-TAT GTA AAT GCT TAT GTA AAC CA-3' and 5'-TAC ATC TAG AAA AAG GAG C-3'; for the 54A0 fragment 5'-TAT GTA AAT GCT TAT GTA AAC CA-3' and 5'-ATC AAA ACT GTG CCG CAG-3'; and for the 0W0 fragment 5'-CTG CAG AAG CTT GGT CCC-3' and 5'-ACA GGA TGT ATA TAT CTG-3'. The PCR was purified by ion exchange chromatography using a 1.8 ml SOURCE 15Q (GE Healthcare) column.

Nucleosomes were assembled on 54A18 DNA fragments for RSC and SWI/SNF repositioning. Each 10 μl reaction contained 1 pmol of wild-type and mutant nucleosomes assembled on Cy3 and Cy5 labelled DNA, respectively, 50 mM NaCl,

50 mM Tris pH 7.5, 3 mM $MgCl_2$, 1 mM ATP and the quantity of remodeller specified in figures. Samples were incubated in 0.2 ml thin-walled PCR tubes (ABgene, UK) in an Eppendorf mastercycler with heated lid at 30°C for various time points, before reaction termination by transferal to ice and addition of 500 ng of HindIII-digested bacteriophage lambda competitor DNA (Promega, USA) and 5% (w/v) sucrose. Samples were resolved on a native PAGE gel (5% acrylamide:bis acrylamide (49:1 ratio), 0.25x TBE buffer (0.5 mM EDTA, 22.3 mM Tris-borate, pH 8.3), 0.1% APS and 0.1% TEMED). Gels were cast horizontally between 20 by 20 cm glass plates using 1.5 mm Teflon spacers, before mounting vertically in the gel apparatus (Thermo Fischer Scientific, USA) and pre-running at 300 V for 3 h with continuous pump recirculation of 0.2x TBE buffer between the upper and lower compartments at 4°C. Gels were run at 300 V for 3.5 h and imaged using a Phosphoimager FLA-5100 (Fujifilm, Japan). Gel band intensities were quantitated using AIDA software (Raytest, Germany) and the remodeller repositioning at each time point calculated from the intensity of the sum of all end positions relative to the sum of the major initial and all end positions. The initial rate was calculated as previously described (Ferreira et al., 2007). Each initial rate was repeated at least three times using chromatin prepared in separate assembly reactions.

Dimer exchange assays

Histone H2A T10C was fluorescently labelled by a Cy5 mono maleimide dye (GE Healthcare). Donor nucleosomes were produced by assembly of tetramers and Cy5 labelled dimers onto Cy3 labelled 54A18 DNA fragments. To measure nucleosome assembly efficiency 2 pmol of each assembly reaction was resolved by native PAGE and the assembly quantified by measuring the summed intensity of all nucleosome bands relative to 1 pmol of Cy3 labelled 54A18 DNA. Each 10 µl reaction contained 0.25 pmol of donor nucleosome, 0.75 pmol (3 fold excess) wild-type tetrasome acceptor assembled on 0W0 DNA fragments, 50 mM NaCl, 50 mM Tris pH 7.5, 3 mM $MgCl_2$, 1 mM ATP and the quantity of remodeller specified in figure 6. Reactions were incubated in an Eppendorf mastercycler with heated lid at 30°C for the specified times. Reactions were terminated by transfer to ice and the addition of 500 ng of HindIII-digested bacteriophage lambda competitor DNA (Promega, USA) and 5% (w/v) sucrose. Samples were resolved on a native PAGE gel and the

percentage of dimer transfer for each time point was calculated from the intensity of the acceptor relative to the total Cy5 donor and acceptor fluorescence. The data was adjusted to give 0% transferred at time 0. All experiments were repeated at least three times using different nucleosome assemblies.

Supplemental References

Anderson, J. D., Thastrom, A., and Widom, J. (2002). Spontaneous access of proteins to buried nucleosomal DNA target sites occurs via a mechanism that is distinct from nucleosome translocation. Mol Cell Biol *22*, 7147-7157.

Chandy, M., Gutierrez, J. L., Prochasson, P., and Workman, J. L. (2006). SWI/SNF displaces SAGA-acetylated nucleosomes. Eukaryot Cell *5*, 1738-1747.

Dorigo, B., Schalch, T., Bystricky, K., and Richmond, T. J. (2003). Chromatin fiber folding: requirement for the histone H4 N-terminal tail. J Mol Biol *327*, 85-96.

Ferreira, H., Flaus, A., and Owen-Hughes, T. (2007). Histone modifications influence the action of Snf2 family remodelling enzymes by different mechanisms. J Mol Biol *374*, 563-579.

Flaus, A., and Richmond, T. J. (1998). Positioning and stability of nucleosomes on MMTV 3'LTR sequences. J Mol Biol *275*, 427-441.

Huynh, V. A., Robinson, P. J., and Rhodes, D. (2005). A method for the in vitro reconstitution of a defined "30 nm" chromatin fibre containing stoichiometric amounts of the linker histone. J Mol Biol *345*, 957-968.

Lowary, P. T., and Widom, J. (1998). New DNA sequence rules for high affinity binding to histone octamer and sequence-directed nucleosome positioning. J Mol Biol *276*, 19-42.

Luger, K., Rechsteiner, T. J., and Richmond, T. J. (1999). Preparation of nucleosome core particle from recombinant histones. Methods Enzymol *304*, 3-19.

Park, Y. J., Dyer, P. N., Tremethick, D. J., and Luger, K. (2004). A new fluorescence resonance energy transfer approach demonstrates that the histone variant H2AZ stabilizes the histone octamer within the nucleosome. J Biol Chem *279*, 24274-24282.

Philo, J. S. (2006). Improved methods for fitting sedimentation coefficient distributions derived by time-derivative techniques. Anal Biochem *354*, 238-246.

Rackham, O., and Chin, J. W. (2005). A network of orthogonal ribosome x mRNA pairs. Nat Chem Biol *1*, 159-166.

Saha, A., Wittmeyer, J., and Cairns, B. R. (2002). Chromatin remodeling by RSC involves ATP-dependent DNA translocation. Genes Dev *16*, 2120-2134.

Stafford, W. F., 3rd (1992). Boundary analysis in sedimentation transport experiments: a procedure for obtaining sedimentation coefficient distributions using the time derivative of the concentration profile. Anal Biochem *203*, 295-301.
